# CCAAT/enhancer binding protein β directly regulates the expression of the complement component 3 gene in neural cells: implications for the pro-inflammatory effects of this transcription factor

**DOI:** 10.1186/s12974-014-0223-2

**Published:** 2015-01-24

**Authors:** Elena Hernandez-Encinas, Diana Aguilar-Morante, Marta Cortes-Canteli, Jose A Morales-Garcia, Elena Gine, Angel Santos, Ana Perez-Castillo

**Affiliations:** Instituto de Investigaciones Biomédicas, (CSIC-UAM), Arturo Duperier, 4, 28029 Madrid, Spain; Centro de Investigación Biomédica en Red sobre Enfermedades Neurodegenerativas (CIBERNED), 28031 Madrid, Spain; Present address: Laboratory of Neurobiology and Genetics, The Rockefeller University, 1230 York Avenue, New York, NY 10065 USA; Departamento de Bioquímica y Biologia Molecular, Facultad de Medicina, UCM, Plaza Ramón y Cajal s/n, 28040 Madrid, Spain

**Keywords:** C/EBPβ, C3, neuroinflammation, neural cells, transcriptional regulation

## Abstract

**Background:**

The CCAAT/enhancer-binding protein β (C/EBPβ) is a transcription factor, which was first identified as a regulator of differentiation and inflammatory processes mainly in adipose tissue and liver; however, its function in the brain was largely unknown for many years. Previous studies from our laboratory indicated that C/EBPβ is implicated in inflammatory process and brain injury, since mice lacking this gene were less susceptible to kainic acid-induced injury.

**Methods:**

We first performed cDNA microarrays analysis using hippocampal RNA isolated from *C/EBPβ*^+/+^ and *C/EBPβ*^−/−^ mice. Immunocytochemical and immunohistochemical studies were done to evaluate C/EBPβ and C3 levels. Transient transfection experiments were made to analyze transcriptional regulation of *C3* by *C/EBPβ*. To knockdown *C/EBPβ* and *C3* expression, mouse astrocytes were infected with lentiviral particles expressing an shRNA specific for *C/EBPβ* or an siRNA specific for *C3*.

**Results:**

Among the genes displaying significant changes in expression was complement component 3 (*C3*), which showed a dramatic decrease in mRNA content in the hippocampus of *C/EBPβ*^−/−^ mice. C3 is the central component of the complement and is implicated in different brain disorders. In this work we have found that C/EBPβ regulates C3 levels in rodents glial *in vitro* and in the rat *Substantia nigra pars compacta* (SNpc) *in vivo* following an inflammatory insult. Analysis of the mouse C3 promoter showed that it is directly regulated by C/EBPβ through a C/EBPβ consensus site located at position −616/-599 of the gene. In addition, we show that depletion of C/EBPβ by a specific shRNA results in a significant decrease in the levels of C3 together with a reduction in the increased levels of pro-inflammatory agents elicited by lipopolysaccharide treatment.

**Conclusions:**

Altogether, these results indicate that C3 is a downstream target of C/EBPβ, and it could be a mediator of the pro-inflammatory effects of this transcription factor in neural cells.

## Background

CCAAT/enhancer-binding protein β (C/EBPβ), is a member of a family of transcription factors consisting of six structurally related basic leucine-zipper DNA-binding proteins. All six members of this family exhibit a high sequence similarity in their C-terminal DNA binding domain and a more diverge regulatory N-terminus [1,2]. C/EBPβ is encoded by a single mRNA, yet translation of this mRNA results in the synthesis of three C/EBPβ isoforms based on alternative translation initiation sites [3,4]. C/EBPβ is expressed in numerous tissues, including liver, adipose tissue, kidney, lung, ovary, mammary gland, and hematopoietic tissues and regulates a variety of biological processes, including metabolism, proliferation and differentiation, and immune response [1,5-7]. This regulation takes place through the induction or repression of many genes involved in these processes, such as proliferative- or differentiation-related markers, cytokines, and many proinflammatory genes [1,8]. Also, due to its relevance in the indicated cellular processes, C/EBPβ also is involved in the pathogenesis of different diseases, for example, cancer, hyperinflammatory processes, and bacterial infections [1].

Regarding the central nervous system (CNS), it has been shown that the *C/EBPβ* gene is expressed in different areas of adult rodents [9] and different studies, including those from our group, have indicated that C/EBPβ can have important functions in the brain. It has been shown that C/EBPβ plays an important role in the consolidation of long-term memory, suggesting a very important role for this protein in the hippocampus [10,11] and Menard *et al*. have defined a MEK-C/EBP pathway as being essential for the differentiation of cortical progenitor cells into postmitotic neurons [12]. In this regard, we have demonstrated that C/EBPβ serves as a critical factor in neuronal differentiation [13]. More recently we have demonstrated that C/EBPβ regulates the expression of several genes involved in inflammatory processes and brain injury [14] and mice lacking C/EBPβ showed a reduced inflammatory response after kainic acid injection and exhibited a dramatic reduction in pyramidal cell loss in the CA1 and CA3 subfields of the hippocampus [15]. These data reveal an essential function for C/EBPβ in the pathways leading to brain damage and suggest that C/EBPβ should be considered as a therapeutic target in brain injury and neurodegenerative disorders where neuronal cell death and inflammation are involved. Finally, we have also shown that *C/EBPβ* is expressed in the dentate gyrus of the hippocampus and has a key role in regulating the proliferation and differentiation of the stem/progenitor cell population [16].

The complement system plays important roles in the immune response, including chemotaxis, phagocytosis, cell adhesion, and B and T cell differentiation [17,18], and it is a very important line of defense against infections through the elimination of invading pathogens and regulation of the adaptive immune response [19,20]. More recent works show that complement components can be produced by astrocytes, microglia and neurons and that the complement system also plays important roles in the CNS that extend far beyond host defense and inflammatory processes [21-27]. Complement component 3 (C3) is a 185-kDa glycoprotein, which is the central player in the complement cascade and is involved in opsonization of pathogens, inflammatory processes, and immune response [28]. Regarding the brain, C3 has been shown to be essential for the elimination, or pruning, of incorrect synapsis during development [26,29,30]. In fact, mice deficient in C3 exhibit deficits in synaptic remodeling, increased synaptic connectivity, and enhanced epileptiform activity due to failed synaptic pruning [31]. Also, it has been shown that C3 regulates hippocampal neurogenesis in adult mammalian brain [32] and C3-deficient mice present impaired neurogenesis following cerebral ischemia [33,34]. These results point to C3 as a positive regulator of both basal and ischemia-induced neurogenesis.

Besides its role in the CNS in physiological conditions, uncontrolled complement activation in the brain also has been shown to be involved in the neuroimmunological and inflammatory processes that underlie the pathophysiology of many acute and neurodegenerative disorders, including Alzheimer’s, Parkinson’s, dementia, meningoencephalitis, and multiple sclerosis [25,35-42]. Increased levels of C3 have been found in the cerebrospinal fluid (CSF) of patients with Parkinson’s and Alzheimer’s diseases and these levels augment with the progression of the disease [42]. Zanjani *et al*. showed that C3 localized surrounding β-amyloid plaques in early stages of Alzheimer disease, when an important loss of synapsis takes place [43]. Also, enhanced synthesis of different complement components has been reported in animal models of neurodegeneration and neuroinflammation, such as exposure to neurotoxins, virus-induced encephalomyelitis, and excitotoxic kainic acid lesion [44-47].

In this work, we have analyzed the regulation of C3 by C/EBPβ and its possible involvement as a mediator of the effects of C/EBPβ in inflammatory processes. First, we have shown, by microarray and quantitative RT-PCR analysis, a dramatic decrease in C3 mRNA content in the hippocampus of *C/EBPβ* knockout mice. The analysis of the mouse C3 promoter showed that C3 is directly regulated by C/EBPβ, primarily through a C/EBPβ consensus site located at position −616/-599 of the gene. Expression studies showed a parallel induction of C/EBPβ and C3 proteins by lipopolysaccharide (LPS) *in vitro* in primary cultures of astrocytes and microglial cells and *in vivo* in the *Substantia nigra pars compacta* (*SNpc*) of adult rats. Ectopic expression of C/EBPβ in mouse cultured astrocytes induced the expression of the endogenous C3 gene. On the other hand, depletion of C/EBPβ by a specific shRNA diminished C3 expression levels together with a reduction in interleukin-1β (IL-1β) and cyclooxygenase type 2 (COX-2), after LPS treatment. Altogether, these results suggest a direct role of C3 in the pro-inflammatory effects of C/EBPβ.

## Methods

### Animals

Adult male Wistar rats (8 to 12-weeks old) and neonatal Wistar rats (2-days-old) were used throughout the study. Microarrays analysis was performed in adult *C/EBPβ*^+/+^ and *C/EBPβ*^−/−^ mice. *C/EBPβ*^+/+^ and *C/EBPβ*^−/−^ mice were generated from heterozygous breeding pairs, kindly provided by C. M. Croniger and R. W. Hanson (Case Western Reserve University, Cleveland, OH) [48]. Genotypes were identified using genomic PCR, with DNA prepared from tail using the REDExtract-N-AmpTM tissue PCR kit (XNAT kit, Sigma-Aldrich, Madrid, Spain). All procedures with animals were specifically approved by the Ethics Committee for Animal Experimentation of the Instituto de Investigaciones Biomedicas and carried out in accordance with the European Communities Council, directive 86/609/EEC. Special care was taken to minimize pain or discomfort of animals.

### Microarray analysis

Total RNA was extracted from hippocampus of *C/EBPβ*^+/+^ and *C/EBPβ*^−/−^ mice using Trizol (Agilent Technologies, Madrid, Spain) and the integrity of RNA confirmed in an Agilent 2100 Bioanalyzer. The microarray analysis (seven wild type and seven knockouts samples) was performed using the G4122F Whole Mouse Genome Microarray Kit (Agilent Technologies, Madrid, Spain), at the CNIC Genomic Department. The chips were scanned using the Agilent G2567AA Microarray Scanner System (Agilent Technologies, Madrid, Spain). Image analysis and data collection were carried out using the Agilent Feature Extraction 9.1.3.1. (AFE, Agilent Technologies, Madrid, Spain). The AFE algorithm yields a background-adjusted signal by subtracting the background signal from the mean signal.

### Real-time PCR

Total hippocampal RNA samples (2 μg) were used for the synthesis of cDNA by reverse transcription using the Reverse Transcription System (Promega Corporation, Madrid, Spain) with a pd(N)6 random hexamer. Real-time PCR was performed in an ABI Prism machine using the SYBR Green PCR Master Mix (Applied Biosystems, Madrid, Spain) and 300 nM concentrations of specific primer. The primers used for the determination of the concentration of C3 mRNA were: 5’-ACC TTA CCT CGG CAA GTT TCT-3’ (forward sequence) and 5’-TTG TAG AGC TGC TGG TCA GG- 3’ (reverse sequence), which synthesized a fragment of 140 bp. In all runs, melting curves were performed to ensure that only one DNA fragment was amplified. Cycle threshold for wild type mice (dilution 1:10) was around 25. Amplification of the 18S rRNA was used for normalization of cDNA loading in the PCR as previously described [49]. The relative mRNA content was determined with the 2 ^-Δ ΔCt^ method [50].

### Cells culture, gene silencing and lentiviral infection

GL261 murine glioblastoma cells were obtained from the NCI-Frederick Cancer Research Tumor Repository (Frederick, MD) and propagated in RPMI medium with 10% fetal bovine serum as described [51]. Rat and mouse primary astrocyte and microglial cultures were prepared as previously described [52]. Cultures were stimulated with LPS (10 μg/ml) and cells harvested 24 hours later for evaluation of C/EBPβ and C3 content.

For C3 silencing, mouse astrocytes were grown in 60-mm plates and transiently transfected with 150 pmoles of an siRNA specific for C3 (Santa Cruz Biotechnology, Heidelberg, Germany) using Lipofectamine (as indicated by the manufacturer). The control siRNA also was provided by Santa Cruz Biotechnology. After 24 hours of transfection, plates were treated with LPS at a concentration of 10 μg/ml, and 48 hours later plates were washed twice with cold PBS 1× and processed for protein extraction.

To knockdown C/EBPβ expression, mouse astrocytes were infected with lentiviral particles expressing a shRNA specific for C/EBPβ. The interfering sequence was: GAG CGACGAGTACAAGATG [53]. Annealed oligonucleotides were cloned into pGreenPuroTM shRNA lentiviral vector (Cloning and Expression Lentivector; SBI, System Biosciences, Mountain View, CA, USA), according to the manufacter’s protocol, in which shRNA was expressed under the control of the H1 promoter. The control pGreenPuro™ construct with the luciferase shRNA Template provided by System Biosciences was used as control in all the experiments. The lentiviral particles were obtained in HEK293T cells, which were transfected with the appropriate lentiviral expression vectors and the third generation packaging vectors pMD2-G, pMDLg/pRRE, and pRSV-Rev [54]. The medium containing the lentiviruses was recovered, filtered through a 0.45-μm filter and used to infect astrocytes. The infection was repeated 12 h and 24 h later. After 14 h from the last infection, half of the plates of each of the groups were treated with LPS (10 μg/ml) for 24 h after which all plates were processed for extraction of protein.

### Promoter cloning and luciferase assay

A fragment of the mouse C3 promoter, from −1624/+87, was PCR-amplified from mouse genomic DNA using specific primers and high-fidelity Eppendorf Triple Master PCR System (Eppendorf, Hamburg, Germany). The primers used were: 5’-GGA GAA CAA TAC AGA GAG GAG G-3’ (forward sequence) and 5’-AGT GAA GGA AAA AGG TGG AAG-3’ (reverse sequence). The entire promoter fragment, P.C3/1711 was sequenced and subcloned in the promoterless luciferase reporter vector pGL4.10. Also, two shorter fragments (−255/+42; P.C3/297) and −79/+42; P.C3/121) were PCR amplified from the P.C3/1711 fragment. Point mutations were generated by PCR using specific mutated primers and the Quick Change Site-Directed Mutagenesis Kit (Agilent Technologies, Madrid, Spain). This system uses the Pfu Turbo DNA Polymerase and subsequent digestion with DpnI, to eliminate the methylated parental band. Mutations were confirmed by sequencing. Mutated primers were: P.C3/MutA: 5’-AAG TAG GGC *CCG* GGT *CCT* GCC CAG CAA CTG A-3 (for the C/EBPβ binding site located at −616/-599) and P.C3/MutB: 5’-CTT AGG AAA *CGC ACA ATG TCT CAA G*TG GGC AGT CCC-3’ (for the C/EBPβ binding site at positions −108/-94). For transient transfection experiments, semiconfluent GL261 cells were grown in 24-well plates and transfected with lipofectamine 2000 (Invitrogen, Madrid, Spain) using the different constructs described above (1.5 μg DNA/well) in the presence or absence of the C/EBPβ overexpression plasmid pcDNA3-C/EBPβ (2 μg DNA/well). Forty-eight hours after transfection, cells were harvested and the luciferase activity determined using a reporter assay system (Promega Corporation, Madrid, Spain). β-galactosidase was used to determine transfection efficiency.

### Chromatin immunoprecipitation

Primary cultures of mouse astrocytes were treated with LPS (10 μg/ml) and 24 hr later chromatin immunoprecipitation (ChIP) assay was performed, following the manufacturer’s recommendations (Santa Cruz Biotechnology). For immunoprecipitation, the following antibodies were used: 5 μg of rabbit polyclonal anti-C/EBPβ (Santa Cruz Biotechnology) and 2 μg of normal rabbit IgG (Santa Cruz Biotechnology, Mountain View, CA, USA). The precipitated DNA was analyzed by PCR using the following primers: *C/EBPβ*, sense: 5’*-*TGC CCT CAC CCC TTA TCC TCC TC*-*3’; antisense: 5’-GGC AGG TTC AGG AAG GTG GAG AC-3’ (which amplified the C3 promoter from −669 to −525) and GAPDH, sense: 5’-GAG GTC CAC CAC CCT GTT GCT GTA GC -3’; antisense 5’-GCT GAA CGG GAA GCT ACA TGG CAT GG -3’ (which amplified a fragment from +208 to +712). Thermal cycling conditions: one cycle at 95°C for 1 min; 38 cicles at 95°C for 30 s, 60°C for 30 s and 72°C for 30 s; and one cycle at 72°C for 7 min.

### Western blot analysis

Proteins were isolated from cell cultures by standard methods. A total amount of 30 μg of protein was loaded on a 6% or 12% SDS-PAGE gel. After electrophoresis, proteins were transferred to protran nitrocellulose membranes (Sigma-Aldrich, Madrid, Spain), and blots were probed with the indicated primary antibodies, as previously described [14]. The antibodies used were the following: rabbit polyclonal anti-C3 (Abcam, Cambridge, UK), mouse monoclonal anti-C/EBPβ (Abcam, Cambridge, UK), goat polyclonal anti-COX2 (Santa Cruz Biotechnologies, Mountain View, CA, USA), rabbit polyclonal anti-IL1β (Abcam, Cambridge, UK), rabbit polyclonal anti-α-tubulin (Sigma-Aldrich, Madrid, Spain) and secondary peroxidase conjugated antibodies (Jackson Immunoresearch, West Grove, PA, USA). Quantification was performed using Scion Image software (Scion Corporation, http://scion-image.software.informer.com/).

### Nitrites measurement

Accumulation of nitrites in media was assayed by the standard Griess reaction. After 24 hours of transfection with 150 pmoles of an siRNA specific for C3 or a nontargeting siRNA, plates were treated with LPS at a concentration of 10 μg/ml, and 48 hours later supernatants were collected and mixed with an equal volume of Griess reagent (Sigma-Aldrich, Madrid, Spain). Samples were then incubated at room temperature for 15 minutes and absorbance read using a plate reader at 492/540 nm.

### Immunocytochemistry

Cells were processed for immunocytochemistry at the end of the treatment period as previously described [55]. Briefly, primary glial cultures grown on glass cover slips were washed with PBS, fixed for 30 min with 4% paraformaldehyde at 25°C and permeabilized for 30 min with 0.1% Triton X-100 at 37°C. After 1 h incubation with the corresponding primary antibody, cells were washed with PBS and incubated with Alexa-488 or Alexa-647 secondary antibodies for 45 min at 37°C. Then, 6-diaminidine-2-phenylindole (DAPI) was used to stain nuclei. Images were acquired using a Nikon eclipse 90i microscope, equipped with a DS-Fi1 digital camera (Nikon, Amsterdam, Holland). Microscope settings were adjusted to optimize signal-to-noise ratios. To compare fluorescence signals from different preparations, settings were fixed for all samples within the same analysis. The following primary antibodies were used: rabbit polyclonal anti-C3 (Abcam, Cambridge, UK), mouse monoclonal anti-C/EBPβ (Abcam, Cambridge, UK).

### Lipopolysaccharide injection *in vivo*

Adult male rats were anesthetized by intraperitoneal injection of ketamine (60 mg/kg) and medetomidine (0.125 mg/kg) and positioned in a stereotaxic apparatus (Kopf Instruments, Tujunga, CA, USA). LPS (10 μg in 2.5 μl PBS) was injected into the right side of the SNpc (coordinates from Bregma: posterior −4.8 mm; lateral +2.0 mm; ventral +8.2 mm, according to the atlas of Paxinos and Watson) with a 10-μl Hamilton syringe. Flow rate (1 μl/min) was kept constant with a motorized syringe pump (BASi, West Lafayette, IN, USA) and the needle was kept in place for 2 min postinjection before being slowly withdrawn. Control animals of the same age were injected with vehicle. Rats were then housed individually to recover and sacrificed 72 h after lesioning with LPS.

### Immunohistochemistry

Rats previously anesthetized were perfused transcardially with 4paraformaldehyde, and brains removed, postfixed and processed for immunohistochemistry as previously described [56]. The following primary antibodies were used: rabbit polyclonal anti-C3 (Abcam, Cambridge, UK), mouse monoclonal anti-C/EBPβ (Abcam, Cambridge, UK), rabbit anti-tyrosine hydroxylase (TH, Chemicon International Inc., Madrid, Spain) and rabbit polyclonal anti-glial fibrillary acidic protein (GFAP, Dako, Glostrup, Denmark). Neurotrace fluorescent Nissl stain (Molecular Probes, Madrid, Spain) and tomato lectin (Vector Labs, Peterborough, UK) were used to stain neurons and microglial cells, respectively. After being mounted with Vectashield (Vector labs, Peterborough, UK), samples were examined with a spectral LSM710 confocal microscope (Zeiss, Madrid, Spain) equipped with a Nikon DS-Qi1Mc digital camera. To compare fluorescence signals from different preparations, confocal microscope settings were fixed for all samples within the same analysis and adjusted to produce the optimum signal-to-noise ratio. Activated glial cells (microglia and astrocytes) expressing C/EBPβ were counted in the *SNpc* on coronal sections doubled immunostained with tomato lectin (microglia) or GFAP (astrocytes) in five well-defined high magnification (×400) fields in five animals/experimental group, using a computer-assisted image analysis software (Soft Imaging System Corp, Lakewood, CO, USA). Double C/EBPβ/tomato-lectin or C/EBPβ/GFAP positive cells counting were expressed as a percentage over the total number of tomato-lectin^+^ or GFAP^+^ cells, respectively.

## Results

### Microarray analysis

The comparison of the hippocampal transcriptoma between wild type and *C/EBPβ* knockout mice rendered 33 genes differentially expressed. Eighteen of these genes are genes that have been related with inflammation and cellular damage (Table 1). Among those genes, we found C3, which is a factor that plays a central role in the function of the complement system. The content of C3 transcripts was markedly reduced in the hippocampus of *C/EBPβ*^−/−^ mice compared with control ones, and subsequently in this work, we pursued the study of the possible role of this gene in the pro-inflammatory and neural damage effects of C/EBPβ.

**Table 1:** **Genes regulated by** ***C/EBPβ*** **involved in immune/inflammatory response**

**Upregulated genes in** ***C/EBPβ*** ^**+/+**^			
**Accession no.**	**Symbol**	**Name**	**Biological processes**
NM_009778	C3	Complement component 3	Complement activation, immune and inflammatory response, response to stress.
NM_013590	Lzp-s	Plysozyme structural	Cell wall macromolecule catabolic processes, defence response to bacterium.
NM_016972	S1c7a8	Solute carrier family (cationic amino acid transporter, y + system), member 8	Integral component of membrane, amino acid transmembrane transporter activity, toxin transporter activity.
NM_009662	Alox 5	Arachidonate 5-lipoxygenase	Defence response, response to stress, immune response, oxidoreductose and dioxygenase activity.
NM_130452	Bbox1	Butyrobetaine (gamma), 2-oxoglutarate dioxygenase 1 (gamma-butyrobetaine hydroxylase)	Carnitine biosynthesis, adrenal gland development, metabolism.
NM_027961.1	Wfdc3	WAP four-disulfide core domain 3	Inflammation, bacterial opsonization, immune responses.
NM_026331	S1c25a37	Solute carrier family 25, member 37	Mitochondrial biogenesis, neurotransmitter transporter.
NM_053262	Dhrs8	Dehydrogenase/reductase (SDR family) member 8	Steroid dehydrogenase activity, androgen catabolic process, lipid metabolic process, oxidation-reduction process.
**Downregulated genes in** ***C/EPBβ*** ^**+/+**^			
**Accession no.**	**Symbol**	**Name**	**Biological process**
NM_009628.2	ADNP	Activity-dependent neuroprotective protein	Neuropeptide signalling pathway, neurological disorders, inflammation.
NM_008596.1	Sypl2	Synaptophysin-like 2	transporter activity, cellular calcium ion homeostasis.
NM_008380	Inhba	Inhibin beta-A	Cytokine-cytokine receptor interaction, growth factor activity, cell cycle arrest, cellular response to cholesterol.
NM_001002927	Penk1	Preproenkephalin 1	Response to stress, neuropeptide signalling pathway, signal transduction.
NM_172479	S1c38a5	Solute carrier family 38, member 5	Amino acid transport across plasma membrane, oxidative stress, antioxidant defence.
NM_010796	Mg11	Macrophage galactose N-acetyl-galactosamine specific lectin 1	Inflammation, macrophage polarization.
NM_008198	Cfb	Complement factor B	Response to stress, complement activation, immune response.
NM_026954	Tusc1	Tum or suppressor candidate 1	Chronic inflammation, cell cycle regulation.
NM_145575	Cald1	Caldesmon 1	Immune response.

### C/EBPβ regulates the activity of the mouse *C3* promoter

**Figure 1:**
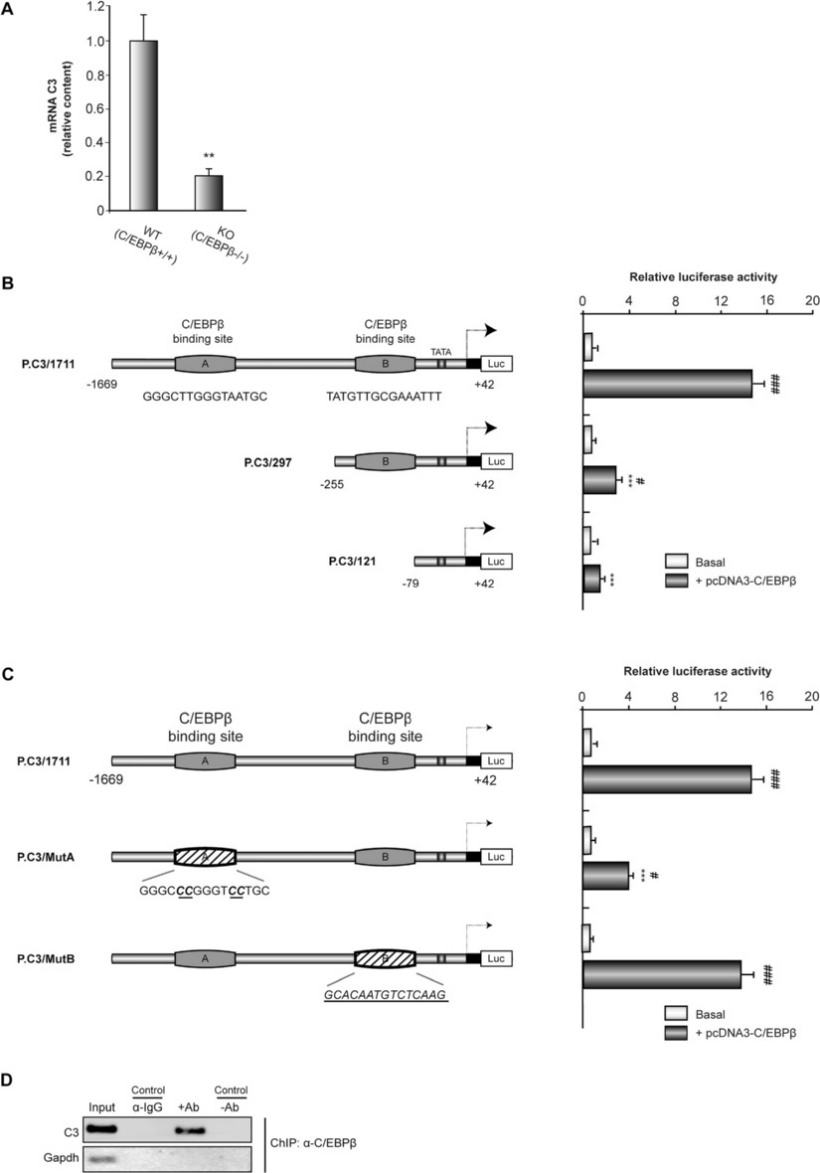
**Regulation by C/EBPβ of the activity of the mouse** ***C3*** **promoter. (A)** RT-PCR analysis of C3 mRNA content in the hippocampus of *C/EBPβ* ^+/+^ and *C/EBPβ* ^−/−^ mice. The graphic shows the mean ± SD of three different experiments. **P <0.01. **(B, C)** Analysis, by transient transfection in GL261 cells, of the effect of different deletions **(B)** and point mutations **(C)** on the activity of *C3* promoter. Data represent the mean ± SD of luciferase activity determined in triplicate in at least three independent experiments. ***P <0.001 *versus* whole **(B)** or non-mutated **(C)** *C3* promoter; #*P* <0.05, ###*P* <0.001 *versus* C3 basal promoter activity. **(D)** Representative image of ChIP analysis of C/EBPβ binding to the endogenous *C3* promoter in primary astrocytes. DNA before immunoprecipitation was used as positive control (Input).

We initially confirmed, by real time PCR, the decrease in C3 mRNA content in the hippocampus of *C/EBPβ*^−/−^ mice indicated by the microarray analysis. Our results showed an 80% decrease in the content of C3 transcripts in the hippocampus of knockout mice compared with controls (Figure 1A). To further substantiate the role of C/EBPβ in regulating *C3* gene expression, we studied whether C/EBPβ regulates C3 promoter activity. First, we performed an *in silico* analysis to search for putative C/EBPβ binding sites in the mouse *C3* promoter. We identified two putative consensus binding sites for this transcription factor located at positions −616/-599 (site A) and −108/-94 (site B). Transient transfection experiments in GL261 cells showed that the cotransfection of P.C3/1711 with C/EBPβ significantly (14-fold) induced the activity of the promoter (Figure 1B), clearly indicating that C3 promoter responds to C/EBPβ, as suggested by the *in silico* analysis. In order to localize the responsive site we first analyzed the response to C/EBPβ overexpression of different deletions of *C3* promoter. When site A was deleted (P.C3/297), the induction by C/EBPβ was drastically reduced. No effect of C/EBPβ was observed when both sites, A and B, were deleted (P.C3/121) (Figure 1B). A more detailed analysis, by point mutation, showed that site A is essential for the regulation by C/EBPβ of the mouse *C3* promoter. When site A was mutated, the induction by C/EBPβ of the promoter activity was reduced from a 14-fold increment in P.C3/1711 to only a 4-fold increase in P.C3/MutA (Figure 1C). In contrast, no effect was observed when site B was mutated (Figure 1C). All these results suggest that the C/EBPβ binding site A is the most important for the regulation by C/EBPβ of the mouse *C3* promoter.

To provide direct evidence that C/EBPβ is recruited to the endogenous *C3* promoter during transcription *in vivo*, we performed standard ChIP assays, which allow the detection of proteins bound to specific regions of DNA *in vivo*. For these assays we used primary cultures of astrocytes treated with LPS and a specific antibody against C/EBPβ to precipitate the complex formed by DNA and this transcription factor. ChIP analysis with C/EBPβ antibody showed binding of this transcription factor to the *C3* promoter at the C/EBPβ consensus binding site A (Figure 1D). As a control of specificity of the assay, binding of C/EBPβ to the housekeeping GAPDH locus was almost undetectable. These data indicate that C/EBPβ interacts with the A region of the *C3* promoter.

### Lipopolysaccharide simultaneously induces the expression of *C/EBPβ* and *C3* genes in primary cultures of mouse astrocytes and microglial cells

**Figure 2:**
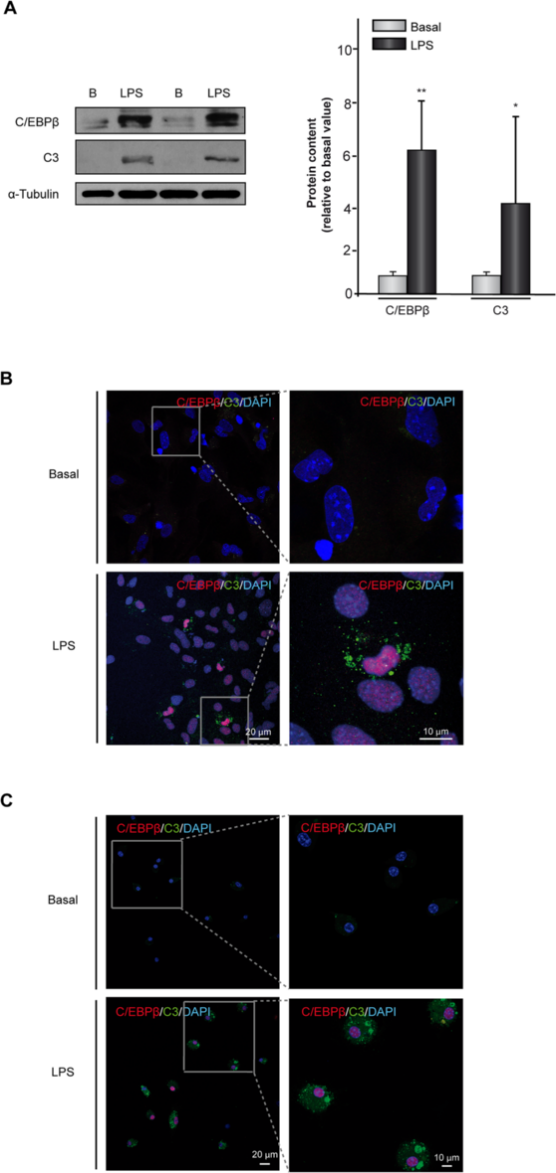
**Induction of** ***C/EBPβ*** **and** ***C3*** **gene expression in mouse astrocytes and microglial cells treated with lipopolysaccharide (LPS).** Astrocytes and microglial cells were cultured and treated with vehicle or LPS (10 μg/ml) and the content of C/EBPβ and C3 proteins determined 24 h later using specific antibodies, as indicated in Methods. **(A)** Representative western blot showing the levels of C/EBPβ and C3. Quantification analysis is shown. Values represent the mean ± SD of five different experiments. *P <0.05, **P <0.01. **(B, C)** Primary astrocytes **(B)** or microglial **(C)** cells were treated as commented above and the expression of C/EBPβ (red) and C3 (green) was evaluated using immunofluorescence techniques, as described in Methods. Nuclei were stained with DAPI.

We have previously shown that pro-inflammatory and cell injury factors induce the expression of C/EBPβ in glial cells [15]. Here, we used the known pro-inflammatory factor LPS to induce the expression of C/EBPβ and simultaneously analyzed the expression of C3 in primary cultures of mouse astrocytes and microglial cells. Western blot analysis showed that in astrocytes LPS induced the expression of both C/EBPβ and C3. As can be seen in Figure 2A, LPS elicited a significant increase in the content of C/EBPβ and C3 (six-fold and four-fold, respectively, compared with non-treated cultures). Immunocytochemistry analysis showed similar results with an increase of C/EBPβ in the nuclei and C3 in the cytosol of astrocytes (Figure 2B). Interestingly, our results clearly show that this induction of C/EBPβ and C3 protein levels by LPS occurs in the same cells (Figure 2B). In the case of microglial cells, a robust induction by LPS of both C/EBPβ and C3 also was observed (Figure 2C).

### Effect of genetic manipulation of C/EBPβ and C3 content on the expression of IL-1β and COX-2 in mouse astrocytes

**Figure 3:**
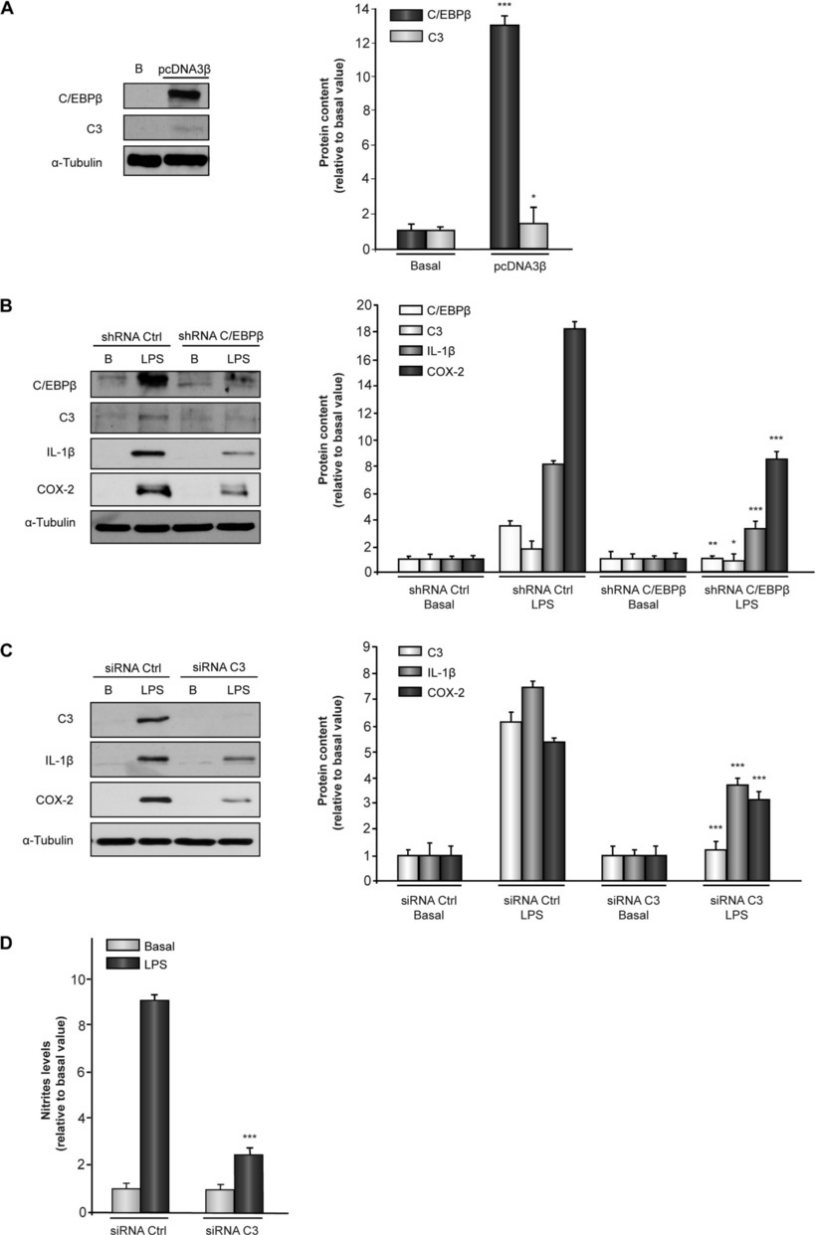
**Effect of C/EBPβ and C3 on** ***IL-1β*** **and** ***COX-2*** **gene expression in mouse astrocytes.** Astrocytes were transiently transfected with the indicated vectors or oligonucleotides, treated or not with lipopolysaccharide (LPS), and 24 h later the content of C/EBPβ, C3, IL-1β and COX2 proteins analyzed as indicated in Methods. **(A)** Representative western blot showing expression of C/EBPβ and C3 in astrocytes overexpressing C/EBPβ (pcDNA3β). Quantification analysis is shown. Data represent the mean ± SD of five different experiments. *P <0.05, ***P <0.001 *versus* basal values. **(B)** Representative western blot showing expression of C/EBPβ, C3, IL-1β, and COX-2 in astrocytes transfected with a C/EBP shRNA and treated or not with LPS. Quantification analysis is shown. Values represent the mean ± SD of five different experiments. *P <0.05, **P <0,01, ***P <0.001 versus shRNA Ctrl. **(C)** Representative Western blot showing expression of C3, IL-1β, and COX-2 in astrocytes transfected with C3 siRNA and treated or not with LPS. Quantification analysis is shown. Values represent the mean ± SD of five different experiments. **(D)** Astrocytes were transfected with C3 siRNA or a non-targeting siRNA (siRNA Ctrl), treated or not with LPS and nitrite production was measured as indicated in Methods. ***P <0.001 *versus* siRNA Ctrl + LPS.

So far, our analysis of C3 regulation by C/EBPβ in glial cells was based in the simultaneous induction of the expression of both genes by LPS. To further analyze the regulation of C3 expression by C/EBPβ and to avoid any C/EBPβ-independent effects of LPS on the expression of C3, we forced the expression of C/EBPβ by transiently transfecting mouse astrocytes with the pcDNA3β vector and analyzed the expression of the endogenous C3 gene. As shown in Figure 3A the content of C/EBPβ is clearly increased in transfected cells and simultaneously a two-fold increase in C3 was observed. In contrast, when the LPS-induced increase of C/EBPβ was decreased by RNA interference with an shRNA specific for this transcription factor a clear reduction in the induction of C3 gene expression was observed (Figure 3B). Moreover, IL-1β and COX2, two well-known inflammatory mediators, which are induced by C/EBPβ [15] also were decreased in the presence of C/EBPβ shRNA (Figure 3B). Furthermore, when the LPS induction of C3 was blocked by a specific siRNA, a clear decrease in IL-1β and COX-2 also was observed (Figure 3C), suggesting that C3 could play a role in the induction of these inflammatory agents by C/EBPβ.

### Lipopolysaccharide simultaneously induces the expression of *C/EBPβ* and *C3* genes in primary cultures of rat astrocytes and microglial cells

**Figure 4:**
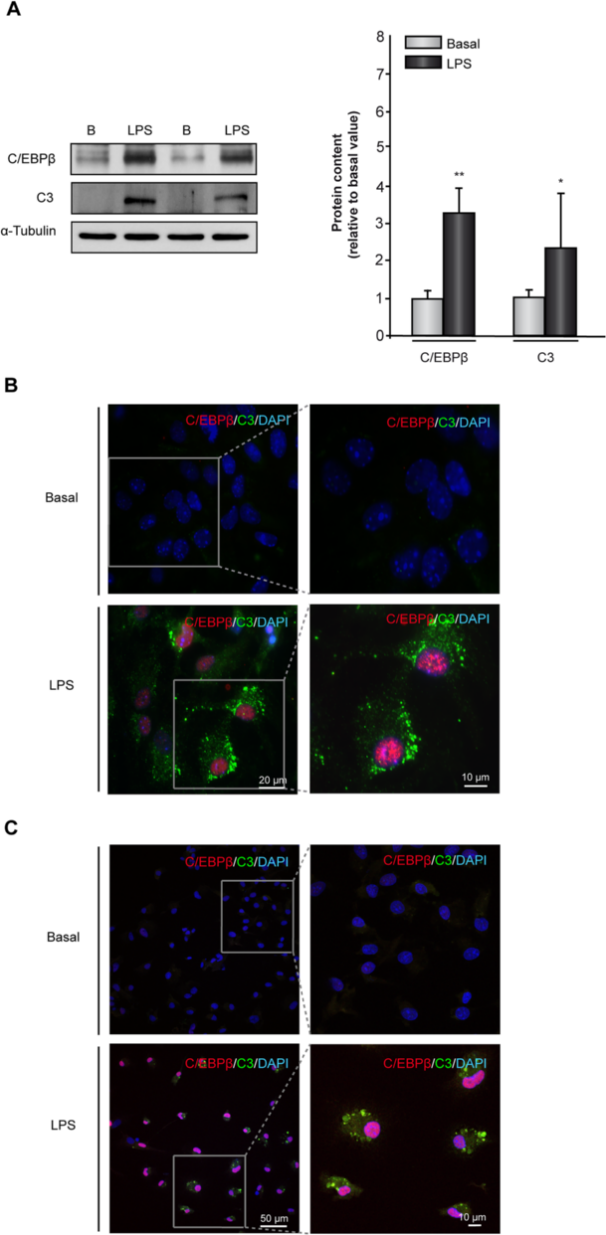
**Induction of** ***C/EBPβ*** **and** ***C3*** **gene expression in rat astrocytes and microglial cells treated with lipopolysaccharide (LPS).** Astrocytes and microglial cells were cultured and treated with vehicle or LPS (10 μg/ml) and the content of C/EBPβ and C3 proteins determined 24 hours later using specific antibodies, as indicated in Methods. **(A)** Representative western blot showing the levels of C/EBPβ and C3. Quantification analysis is shown. Values represent the mean ± SD of five different experiments. *P <0.05, **P <0.01. **(B, C)** Primary astrocytes **(B)** or microglial **(C)** cells were treated as commented above and the expression of C/EBPβ (red) and C3 (green) was evaluated using immunofluorescence techniques, as described in Methods. Nuclei were stained with DAPI.

Next, we analyzed whether the effect of LPS on the expression of C/EBPβ and C3 observed in mice cultures of glial cells also was detected in rat primary cultures of astrocytes and microglial cells. As shown in Figure 4, yet again a parallel increase in cell content of C/EBPβ and C3 was observed. The western blot results showed an approximately three- and two-fold increase in C/EBPβ and C3 content, respectively, in astrocytes (Figure 4A). Immunocytochemistry analysis also showed a coincident increase of C/EBPβ in the cell nuclei and C3 in the cytosol of both astrocytes and microglial cells, as previously observed in mice glial cultures, with a clear colocalization of both proteins in the same cells (Figure 4B-C).

### Effect of lipopolysaccharide injection on the expression of *C/EBPβ* and *C3* genes *in vivo* in the *SNpc* of adult rats

**Figure 5:**
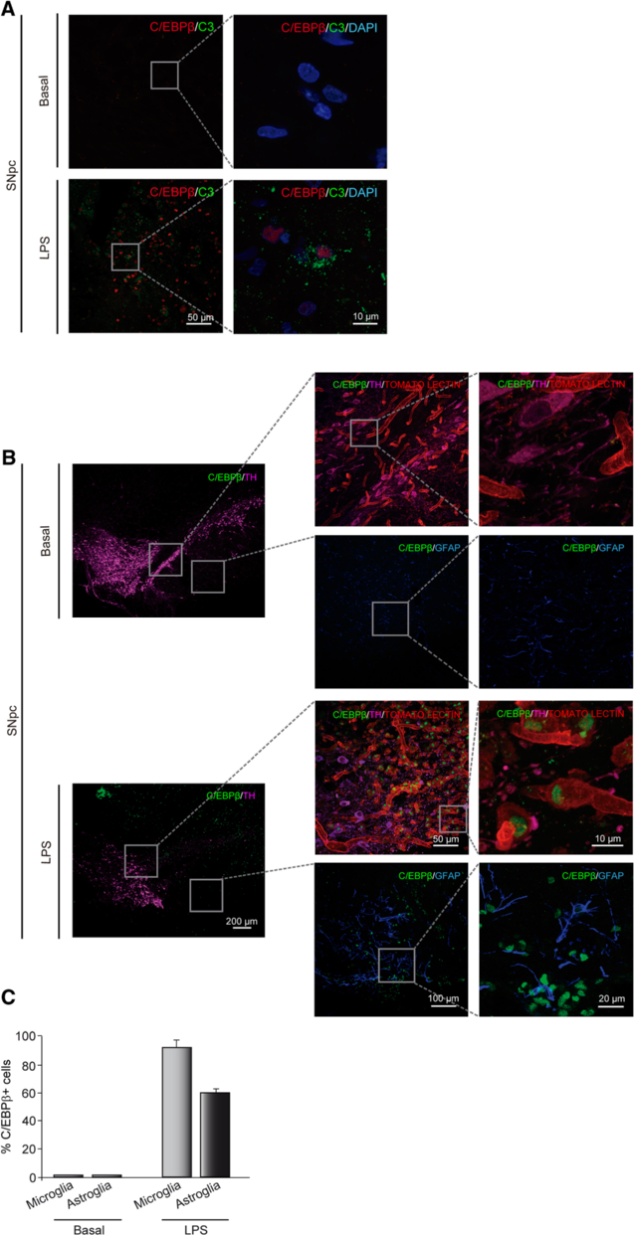
**Effect of lipopolysaccharide (LPS) on the expression of C/EBPβ and C3 in the** ***SNpc*** **of the rat.** Rats were injected with LPS in the *SNpc* and 72 hours later the expression of C/EBPβ and C3 was determined as indicated in Methods. **(A)** Immunohistochemistry analysis of coronal brain sections showing C/EBPβ and C3 expression in the *SNpc* of adult rats injected or not with LPS. Labeling was performed using specific antibodies recognizing C/EBPβ (red) and C3 (green). Nuclei were stained with DAPI (blue). **(B)** Immunohistochemistry analysis of coronal brain sections showing C/EBPβ and TH expression in astrocytes and microglia cells in the *SNpc* of adult rats injected or not with LPS. Labeling was performed with tomato lectin (to identify microglial cells) and specific antibodies recognizing GFAP (astrocytes, blue), C/EBPβ (green) and TH (magenta). **(C)** Quantification of the number of double stained C/EBPβ/tomato-lectin^+^ and C/EBPβ/GFAP^+^ cells in the *SNpc*. Values are expressed as a percentage of double C/EBPβ/tomato-lectin^+^ and C/EBPβ/GFAP^+^ inmmunoreactive cells with respect to the total number of microglial or astroglial cells, respectively.

In order to further extend the studies realized in glial primary cell cultures, we analyzed the effect of LPS injection on C/EBPβ and C3 levels in rats *in vivo*. For this purpose, adult rats were injected with either vehicle or LPS into the *SNpc*. This brain area, in contrast with others (such as the hippocampus), is very sensitive to the action of LPS. Our results show that the injection of LPS noticeable increased the expression of C/EBPβ and C3 in the damaged *SNpc* (Figure 5A) and that the induction of the expression of these two proteins took place in the same cells. To identify the cell type specificity of this activation, we next performed double labeling using tomato lectin, anti-TH, and anti-GFAP to identify microglia, dopaminergic cells, and astrocytes, respectively. C/EBPβ was mostly expressed in microglial cells. Our results show that microglia accumulated within the damage area, very close to the dopaminergic neurons, and that they expressed high levels of C/EBPβ. In the case of astrocytes (GFAP^+^ cells), we found C/EBPβ expressing cells in the periphery of the damaged area (Figure 5B). Quantification analysis (Figure 5C) shows a higher amount of microglial cells expressing C/EBPβ compared to astrocytes. These results differ slightly from those obtained in *in vitro* cultures of glial cells where a less number of microglial cells expressed C/EBPβ, compared to the number of astrocytes expressing this transcription factor.

## Discussion

In this work, we have shown that the transcription factor C/EBPβ directly regulates the expression of the *C3* gene, and that this control could be relevant for the pro-inflammatory effects of this transcription factor. By microarray analysis and RT-PCR we showed that the hippocampal content of C3 transcripts was depleted in *C/EBPβ*^−/−^ mice. The analysis of the *C3* promoter showed that this gene was directly induced by C/EBPβ through a C/EBPβ consensus site located at −616/-599 position from the transcription start site. In accordance with these data, LPS induced the expression of C3 in glial cells, at least in part, through the induction of C/EBPβ since the repression of LPS-induction of C/EBPβ by shRNA interference blocked C3 increase. On the contrary, C/EBPβ overexpression by transient transfection induced C3 expression. Additionally, treatment of these cultures with LPS induced the levels of the pro-inflammatory factors IL-1β and COX-2, which were significantly reduced in those cells depleted of C/EBPβ and C3. Finally, we showed that *in vivo*, LPS also induced the expression of both C/EBPβ and C3 in the rat *SNpc*, an LPS-sensitive area, and that both proteins colocalized in the same cells. Collectively, our findings suggest that C3 could be a mediator of the pro-inflammatory action of C/EBPβ.

Regarding the microarray analysis we found 33 genes, out of 2,463 genes included in the analysis, for which expression was altered in the hippocampus of *C/EBPβ*^−/−^, and 18 of these were genes related with inflammatory or cell injury processes (Table 1). The component 3 of the complement was the gene that expression was most markedly decreased in the hippocampus of *C/EBPβ*^−/−^ mice (Table 1). Interestingly, in a previous microarray analysis performed by our group using the neuroblastoma cell line TR overexpressing C/EBPβ, we also identified *C3* as a gene regulated by C/EBPβ [14].

The analysis of the mouse *C3* promoter showed that the relevant site for its regulation by C/EBPβ was the distal C/EBPβ binding site (−616/-599). When this site was deleted (P.C3/297) or more importantly mutated (P.C3/MutA), a drastic reduction in the induction by C/EBPβ of the *C3* promoter activity was observed (from a 14-fold induction in P.C3/1711 to a modest 4-fold induction in P.C3/297 and P.C3/MutA). In contrast, no change in C/EBPβ activation of the *C3* promoter was observed when the proximal site was mutated. The human *C3* promoter has been extensively analyzed regarding its regulation by inflammatory signal, such as interleukin 1 and 6, and viral infection [57-60]. Consistent with our results, these studies suggest that the C/EBP proteins play an important role in the regulation of *C3* gene expression. Specifically, C/EBPβ plays a critical role in the induction by IL-1β and the repression by the hepatitis C virus of *C3* gene expression [59,60]. However, and in contrast with our studies, the region of the human *C3* promoter involved in C/EBPβ action was located in more proximal sequences, −131/-93 [59] and −127/-70 [60] from the transcription start site. Most importantly, in this work we found, in a physiological setting such as primary astrocyte cultures treated with LPS, that the C/EBPβ protein is enriched in the promoter region expanding from −669 to −525 of the endogenous *C3* gene, which is the region implicated in the regulation by C/EBPβ.

In accordance with the regulation of the *C3* promoter by C/EBPβ we showed that LPS strongly induces the expression of *C/EBPβ* and *C3* genes in primary cultures of mouse and rat astrocytes and microglial cells. Both proteins colocalized in the same cells, being C/EBPβ located in the nucleus and C3 found in the cytoplasm. These results are in agreement with previous works, including our reports, showing that in primary cultures of glial cells, LPS induces the expression of C/EBPβ [15,61] and also the expression of C3 [62,63]. However, these authors did not study the regulation of both genes together nor did they study colocalization in the same cells. Also, the results presented here are consistent with a previous report [64] showing a parallel induction by LPS of C/EBPβ and C3 mRNAs in mouse astrocytes, suggesting that C/EBPβ could play an important role in LPS induction of *C3* gene expression. The notion that C3 is regulated by C/EBPβ under inflammatory conditions is further supported by our data showing that the increase in both proteins, as commented above, occurs in the same cells and that the induction of C3 by LPS was abolished when the expression of C/EBPβ is blocked by a specific shRNA. Moreover, the overexpression of C/EBPβ, by transient transfection, induces the expression of the C3 gene in mouse astrocytes in the absence of LPS.

C/EBPβ plays an important role in mediating the effect of diverse pro-inflammatory stimuli, including LPS [65], as a consequence of its induction of pro-inflammatory genes such as IL-1β and COX-2, among others. In agreement with this we showed here that when the LPS-induced increase in C/EBPβ was inhibited in astrocytes by a C/EBPβ specific shRNA, a marked decrease in IL-1β and COX-2 induction was observed. In line with these data, we have previously shown a marked decrease in the induction of IL-1β and COX-2 elicited by LPS in microglial cells from *C/EBPβ*^−/−^ mice [15]. In addition, when the induction of C3 by LPS was specifically inhibited by a specific C3 siRNA, a parallel reduction was observed in the LPS-induced increase of IL-1β and COX-2 levels, suggesting that C3 could play an important role as a mediator of the proinflammatory effects of C/EBPβ in neural cells. Consistent with this notion, a neuroinflammatory role of C3 in different models of brain injury has been shown [66,67]. In addition to the well-described pro-inflammatory effects of C/EBPβ, this transcription factor also regulates many relevant cellular functions, including proliferation, differentiation, metabolism, and immune response [68]. In this regard, it is interesting to point out that treatment of human microglial cultures with macrophage-colony stimulating factor enhances proliferation and a change in the morphology of these cells (towards a less activated phenotype, which is accompanied by a parallel increase in C/EBPβ levels [69]). These results are somehow in contrast with the data presented here clearly showing a pro-inflammatory effect of this transcription factor. Since it is widely accepted the pro-inflammatory effects of C/EBPβ, and specifically its effects on microglial cells [14,15,70,71], this apparent discrepancy may be due to the different properties of human *versus* rodent microglia [72].

Finally, we have demonstrated for the first time an induction of C3 and C/EBPβ protein levels *in vivo* after treatment with LPS. Our results show that the *in vivo* injection of LPS into the *SNpc* of adult rats, a brain area known to be sensitive to the neurodegenerative effects of LPS, results in a strong induction of both C/EBPβ and C3 in the glial cells found in the *SNpc* and in adjacent areas. Importantly, both proteins colocalized in the same cells, further supporting the notion that C/EBPβ regulates C3 expression during inflammatory processes.

## Conclusions

In summary, our results clearly show that *C3* gene expression is under the direct regulation of the transcription factor C/EBPβ in activated glial cells and suggest that C3 could play an important role in the pro-inflammatory effect of this transcription factor in the brain.

## Abbreviations

C/EBPβ: CCAAT/enhancer-binding protein β
ChIP: Chromatin immunoprecipitation
CNS: Central nervous system
COX-2: Cyclooxygenase type 2
CSF: cerebrospinal fluid
C3: Complement component 3
DAPI: 6-diaminidine-2-phenylindole
GFAP: Glial fibrillary acidic protein
IL-1β: Interleukin-1β
LPS: Lipopolysaccharide
SNpc: Substantia nigra pars compacta
TH: Tyrosine hydroxylase
